# *Smad4* is essential for epiblast scaling and morphogenesis after implantation, but nonessential before implantation

**DOI:** 10.1242/dev.202377

**Published:** 2024-06-05

**Authors:** Robin E. Kruger, Tristan Frum, A. Sophie Brumm, Stephanie L. Hickey, Kathy K. Niakan, Farina Aziz, Marcelio A. Shammami, Jada G. Roberts, Amy Ralston

**Affiliations:** ^1^ Cell and Molecular Biology Ph.D. Program, Michigan State University, East Lansing, MI 48824, USA; ^2^ Reproductive and Developmental Sciences Training Program, Michigan State University, East Lansing, MI 48824, USA; ^3^ Department of Biochemistry and Molecular Biology, Michigan State University, East Lansing, MI 48824, USA; ^4^ Human Embryo and Stem Cell Laboratory, The Francis Crick Institute, London NW1 1AT, UK; ^5^ Research Technology Support Facility, Michigan State University, East Lansing, MI 48824, USA; ^6^ The Centre for Trophoblast Research, Department of Physiology, Development and Neuroscience, University of Cambridge, Cambridge CB2 3EG, UK; ^7^ Wellcome Trust – Medical Research Council Stem Cell Institute, University of Cambridge, Jeffrey Cheah Biomedical Centre, Puddicombe Way, Cambridge CB2 0AW, UK; ^8^ Epigenetics Programme, Babraham Institute, Cambridge CB22 3AT, UK; ^9^ Genetics and Genome Sciences Ph.D. Program, Michigan State University, East Lansing, MI 48824, USA; ^10^ Molecular, Cellular, and Integrative Physiology Ph.D. Program, Michigan State University, East Lansing, MI 48824, USA

**Keywords:** PSMAD1/5/9, Extra-embryonic, Maternal and zygotic gene deletion, Epiblast, Morphogenesis, Mouse

## Abstract

Bone morphogenic protein (BMP) signaling plays an essential and highly conserved role in embryo axial patterning in animal species. However, in mammalian embryos, which develop inside the mother, early development includes a preimplantation stage, which does not occur in externally developing embryos. During preimplantation, the epiblast is segregated from extra-embryonic lineages that enable implantation and development *in utero*. Yet, the requirement for BMP signaling is imprecisely defined in mouse early embryos. Here, we show that, in contrast to previous reports, BMP signaling (SMAD1/5/9 phosphorylation) is not detectable until implantation when it is detected in the primitive endoderm – an extra-embryonic lineage. Moreover, preimplantation development appears to be normal following deletion of maternal and zygotic *Smad4*, an essential effector of canonical BMP signaling. In fact, mice lacking maternal *Smad4* are viable. Finally, we uncover a new requirement for zygotic *Smad4* in epiblast scaling and cavitation immediately after implantation, via a mechanism involving FGFR/ERK attenuation. Altogether, our results demonstrate no role for BMP4/SMAD4 in the first lineage decisions during mouse development. Rather, multi-pathway signaling among embryonic and extra-embryonic cell types drives epiblast morphogenesis postimplantation.

## Teaser

**Summary:** Gene expression, gene deletion and pathway visualization show that *Smad4*-dependent signaling is first active after mouse embryo implantation, when it promotes epiblast morphogenesis and scaling, relative to the whole embryo.

## INTRODUCTION

In animal embryos, including mice, frogs, fish and flies, the bone morphogenic protein (BMP) signaling pathway oversees crucial patterning events early in development. In non-mammalian species, BMP signaling is essential for specification of the dorsal/ventral axis of the early embryo (De Robertis and Sasai, 1996; O'Connor et al., 2006; Zinski et al., 2018). However, the mammalian embryo has an additional developmental task immediately following fertilization: specification of the extra-embryonic lineages that will give rise to placenta and yolk sac and enable development within the mother. Published studies support roles for BMP signaling in extra-embryonic lineage specification before implantation and in subsequent axial patterning, which occurs after implantation (Chu et al., 2004; Graham et al., 2014; Reyes de Mochel et al., 2015; Senft et al., 2019; Sirard et al., 1998; Yamamoto et al., 2009). However, differences in technical approaches used, as well as challenges intrinsic to mouse, have limited origination of a universally accepted model of the role of BMP signaling in mouse embryos throughout pre- and post-implantation stages.

BMP is one of several related and highly conserved molecular signaling pathways belonging to the transforming growth factor beta (TGFβ) superfamily of cytokines. The molecular mechanisms of TGFβ signaling have been carefully studied (Chang, 2016; Massagué and Sheppard, 2023). BMP proteins, like other members of the TGFβ pathway, are secreted ligands that elicit cellular responses by binding to heterodimeric, transmembrane serine-threonine kinase receptors. The activated receptor complex then phosphorylates members of a family of intracellular effectors known as receptor-associated SMADs (R-SMADs). Phosphorylation of R-SMADs allows their association with a co-factor SMAD (co-SMAD) and accumulation in the nucleus, where they impact chromatin and transcription (Hill, 2016). In mammals, R-SMAD activity is encoded by several Smad paralogues, with SMAD1, SMAD5 and SMAD9 (also known as SMAD8) primarily transducing BMP signals, and SMAD2 and SMAD3 primarily transducing Nodal, activin and TGFβ. Remarkably, the mammalian genome encodes a single co-SMAD, SMAD4, which is shared by BMP, Nodal, Activin, and TGFβ signaling pathways.

Across species, BMP signaling has been visualized in embryos using antibodies that specifically recognize the phosphorylated form of the BMP-responsive R-SMADs. This approach has been used to observe gradients of BMP signaling activity that correspond with the dorsal/ventral axis in fly, fish and frog embryos (Dorfman and Shilo, 2001; Plouhinec and De Robertis, 2009; Schohl and Fagotto, 2002; Tucker et al., 2008). In mouse, no graded pSMAD1/5/9 pattern has been reported. Rather, pSMAD1/5/9 is reportedly detected in all cell types of the embryo throughout preimplantation (Graham et al., 2014; Reyes de Mochel et al., 2015). After implantation, pSMAD1/5/9 is detected within a subdomain of extra-embryonic cells, and not within the embryo itself, until it is detected in primordial germ cells and emerging mesoderm during gastrulation (Senft et al., 2019). These observations suggest fundamental differences in the roles of BMP signaling between mammalian and non-mammalian animal embryos, but raise the need for additional, functional lines of evidence.

In mice, individual members of the BMP signaling pathway have been knocked out, but all appear to be dispensable before embryonic day (E) 6.5. Knockout of genes encoding the predominant ligand *Bmp4* (Lawson et al., 1999; Winnier et al., 1995), the receptors *Bmpr2* (Beppu et al., 2000), *Bmpr1a* (Mishina et al., 1995), *Actr1a* (Gu et al., 1999), the R-SMADs encoded by *Smad1* (Tremblay et al., 2001) and *Smad5* (Chang et al., 1999), and the co-SMAD *Smad4* (Sirard et al., 1998; Yang et al., 1998, 2002) all point to essential roles for BMP signaling in extra-embryonic mesoderm, extra-embryonic endoderm and germ cell development. Mechanistically, BMP also interacts with Nodal to pattern the visceral endoderm (VE) and specify distal, and then anterior, VE – extra-embryonic cell types that pattern the embryo by specifying the location of the primitive streak (Robertson, 2014; Waldrip et al., 1998; Yamamoto et al., 2009). These events define gastrulation and anterior/posterior axial patterning, processes which therefore rely on BMP signaling. None of these studies reported that BMP signaling loss of function had any effect on development before E5.5. However, the presence of maternal gene products, provided within the oocyte, could complicate interpretation of zygotic knockout phenotypes. Indeed, evidence exists in other species that maternally supplied BMP pathway members are functional (Das et al., 1998; Faure et al., 2000; Kramer et al., 2002; Miyanaga et al., 2002; Zhang et al., 2020). An additional complication is that mouse embryos are particularly challenging to recover between E4.5 and E6.5, and an *in vitro* protocol that robustly recapitulates *in vivo* development during these stages is lacking. For all of these reasons, the roles for BMP signaling during the peri-implantation period are still unclear.

In contrast to peri-implantation, preimplantation embryos are relatively easy to isolate and culture *in vitro*. Accordingly, several studies have examined BMP signaling in preimplantation development. Culturing preimplantation embryos in the presence of small-molecule BMP inhibitors led to decreased numbers and cell cycle rate of extra-embryonic trophectoderm (TE) and primitive endoderm (PrE) cells, as well as changes in expression of lineage-specific transcription factors, including markers of PrE (SOX17, GATA6), TE (CDX2) and inner cell mass/epiblast (ICM/EPI; OCT4) (Graham et al., 2014; Reyes de Mochel et al., 2015; Stuart et al., 2019). Some of these observations were recapitulated following microinjection of siRNA against *Bmp4* or overexpression of dominant-negative forms of *Bmpr2* (Graham et al., 2014)*.* Overexpression of dominant-negative *Smad4* reportedly phenocopied loss of the upstream signaling components. In principle, these approaches could interfere with the activities of both maternally and zygotically expressed signaling components and thereby achieve more complete loss of function than zygotic null embryos. However, in these manipulated embryos, pSMAD1/5/9 was not examined, and so the extent to which these manipulations disrupted BMP signaling was not directly tested. Moreover, inhibitors are prone to off-target effects, which could further confound interpretation of results (Lowery et al., 2016).

In the present study, we visualize pSMAD1/5/9 in wild-type embryos and in embryos in which *Bmp4* has been maternally and zygotically deleted. We evaluate their lineage specification and morphogenesis throughout preimplantation, peri-implantation and early post-implantation stages*.* We report that, in contrast to previous studies, BMP signaling is apparently dispensable during mouse preimplantation development. However, we describe a novel role for SMAD4-mediated signaling in limiting FGF/ERK signaling to enable the timely execution of EPI morphogenetic events shortly after implantation.

## RESULTS

### Phosphorylated SMAD1/5/9 is first detectable in peri-implantation embryos

**Fig. 1.**
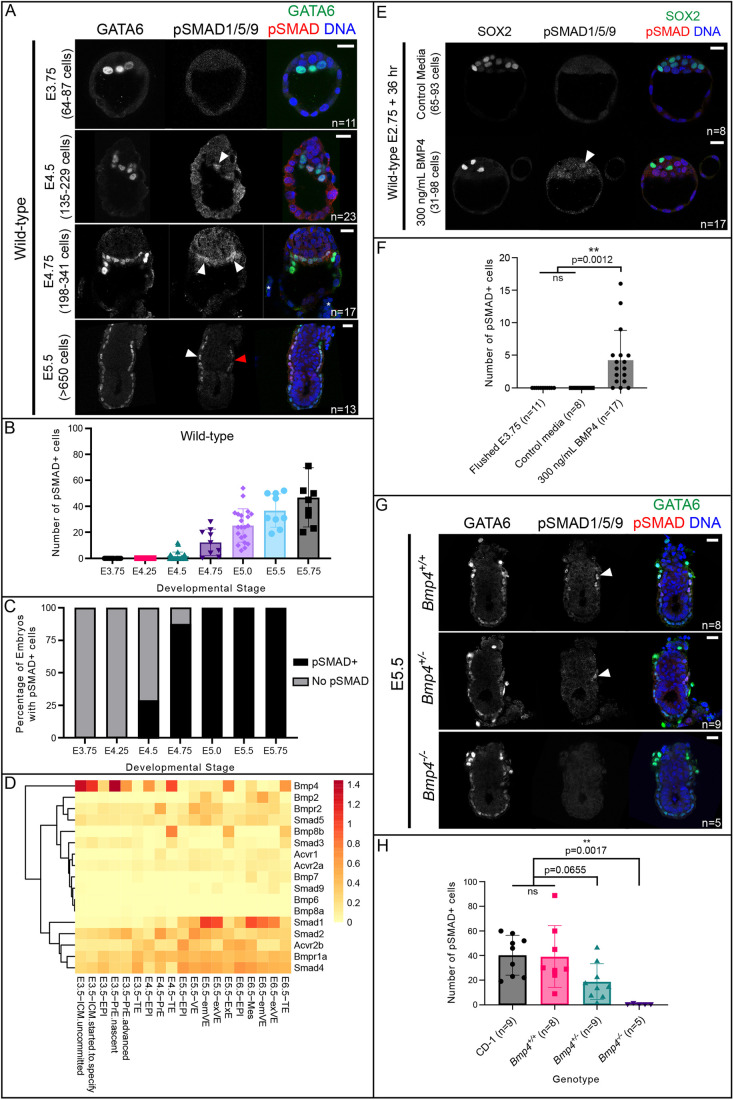
**BMP signaling becomes active in the primitive endoderm lineage at implantation.** (A) SMAD1/5/9 phosphorylation (pSMAD1/5/9) in wild-type CD-1 embryos at E3.75, E4.5, E4.75 and E5.5. In all cases, positive pSMAD1/5/9 signal co-localizes with GATA6 as a marker of PrE and VE. (Asterisks indicate maternal uterine tissue, not part of the embryo shown.) (B) Quantification of total number of pSMAD1/5/9-positive cells in wild-type embryos in A and Fig. S1A. (C) Quantification of the percentage of embryos from A and Fig. S1A that display any pSMAD1/5/9-positive cells versus no pSMAD1/5/9-positive cells. (D) Heat map of the mean normalized expression of BMP pathway genes from scRNA-seq data from Nowotschin et al. (2019). (E) pSMAD1/5/9 in wild-type embryos collected at E2.75 and cultured for 36 h in media containing 300 ng/ml exogenous BMP4. (F) Quantification of the total number of pSMAD1/5/9-positive cells in embryos from E revealed significantly more pSMAD1/5/9-positive cells in BMP4-treated embryos. (G) pSMAD1/5/9 staining is absent in *Bmp4* z null embryos at E5.5. (H) Quantification of total number of pSMAD1/5/9-positive cells in wild-type and *Bmp4* null embryos at E5.5 revealed significantly fewer pSMAD1/5/9-positive cells in *Bmp4* null embryos. White arrowheads indicate positive pSMAD1/5/9 signal. Red arrowhead indicates a GATA6-positive cell, which does not express pSMAD1/5/9. All pairwise comparisons were assessed by one-way analysis of variance (ANOVA) with Tukey's post-hoc test. **P<0.01. ns, not significant. Data are mean±s.d. Scale bars: 10 μm.

To determine when BMP signaling becomes active in the mouse embryo, we first optimized a method to examine the localization of transcription factors SMAD1, 5 and 9, which are phosphorylated in response to ligand/receptor binding (Dijke and Hill, 2004). To achieve this, we used immunofluorescence and an antibody that recognizes phosphorylated SMAD1/5/9 (pSMAD1/5/9; Fig. 1) (Senft et al., 2019; Xu et al., 2019; Yuan et al., 2015) and performed a time course analysis of mouse early embryos. We did not detect pSMAD1/5/9 in preimplantation embryos flushed from uteri between E3.75 and E4.25 (Fig. 1A; Fig. S1A), but pSMAD1/5/9 first became detectable in E4.5 peri-implantation embryos (Fig. 1A). We acknowledge that the assay did not allow us to reliably distinguish possible low levels of pSMAD1/5/9 from background, so we quantified only those cells which were highly pSMAD1/5/9-positive. At E4.5, we detected pSMAD1/5/9 in nuclei of a few ICM cells in 29% of embryos examined (Fig. 1B,C). By E4.75, when embryos have undergone implantation, we observed pSMAD1/5/9-positive cells in 87.5% of embryos evaluated (Fig. 1A-C). Starting at E5.0, we observed pSMAD1/5/9-positive cells within 100% of embryos examined (Fig. 1A-C; Fig. S1A). The observed pSMAD1/5/9 overlapped with a subset of GATA6-expressing PrE (E4.5 and E4.75) and VE (E5.5) cells (Fig. 1A).

To test the specificity of the pSMAD1/5/9 signal, we cultured E5.5 wild-type embryos for 6 h in the presence of LDN-193189 (LDN hereafter), which has been used to disrupt BMP signaling in mouse embryos (Graham et al., 2014; Reyes de Mochel et al., 2015). A concentration of 1 µM LDN was reported as sufficient to inhibit BMP signaling in preimplantation mouse embryos (Reyes de Mochel et al., 2015). However, we found that treatment with 1 µM LDN was highly toxic to embryos at this stage (Fig. S1B). Treatment with 0.25 µM LDN, however, led to complete loss of pSMAD1/5/9 signal in E5.5 embryos (Fig. S1B). Altogether, these observations suggest that BMP signaling becomes active around the time of embryo implantation but is not active during preimplantation stages.

### BMP pathway members are present, but largely inactive, before implantation

A previous report has shown that BMP4 is sufficient to influence gene expression in preimplantation mouse embryos (Goissis et al., 2023), suggesting that preimplantation embryos can respond to exogenous BMP signals. We therefore examined expression dynamics of genes encoding BMP pathway members during preimplantation stages. We analyzed published single-cell RNA-seq (scRNA-seq) data from mouse embryos at stages E3.5-E6.5 (Nowotschin et al., 2019). Many core components of canonical BMP signaling were detectable as early as E3.5, including the ligand *Bmp4*, Type I receptor *Bmpr1a*, Type II receptors *Bmpr2* and *Acvr2b*, receptor-associated SMAD *Smad5* and co-factor SMAD *Smad4* (Fig. 1D; Fig. S2), consistent with blastocyst competence to respond to BMP signals.

Next, we tested whether pSMAD1/5/9 could be induced in preimplantation embryos treated with exogenous BMP4. We cultured compacted eight-cell-stage embryos (E2.75) in 300 ng/ml BMP4 for 36 h to the blastocyst stage (equivalent in cell number to E3.75, as confirmed by cell counts). Although we did not observe pSMAD1/5/9 in any control embryos cultured in unsupplemented medium, we observed low, but detectable, levels of pSMAD1/5/9 in 82% (*n*=14/17) of embryos treated with exogenous BMP4 (Fig. 1E,F). Notably, pSMAD1/5/9 was detected only in the ICM but did not preferentially colocalize with either SOX2-positive (EPI) or SOX2-negative (PrE) cells. The presence of both SOX2-positive and SOX2-negative cells in the ICM is consistent with normal ICM differentiation (Wicklow et al., 2014), in spite of elevated pSMAD1/5/9. Therefore, we conclude that BMP signaling is not normally highly active during preimplantation development, but ICM cells are competent to respond to exogenous BMP signals at these stages, consistent with published investigations (Goissis et al., 2023; Graham et al., 2014; Reyes de Mochel et al., 2015).

We next evaluated pSMAD1/5/9 in embryos shortly after implantation. Consistent with previous reports (Senft et al., 2019; Yamamoto et al., 2009), we detected pSMAD1/5/9 within a zone of the VE that flanks the extra-embryonic ectoderm (EXE) at E5.5 and E5.75 (Fig. 1A; Fig. S1A). This observation is also consistent with evidence that several key components, including *Bmp2*, *Smad1*, *Smad5* and *Bmpr2*, are substantially upregulated around the time of implantation (E4.5-E5.5), particularly in the PrE/VE lineage (Fig. 1D; Fig. S2A,B). Notably, culturing E5.5 embryos in the presence of exogenous BMP4 for 6 h was sufficient to expand the zone of pSMAD1/5/9 within the VE in a dose-dependent manner (Fig. S1C). Thus, the availability of ligand could limit the extent of pathway activation, during both pre- and post-implantation stages.

Finally, we evaluated pSMAD1/5/9 in *Bmp4* null embryos at E5.5. We were unable to detect pSMAD1/5/9 in *Bmp4* null embryos, although it was observed at wild-type levels and localization in homozygous wild-type littermate controls (Fig. 1G,H). In *Bmp4* heterozygous embryos, we observed an intermediate phenotype, where pSMAD1/5/9 was detectable in an intermediate number of cells (Fig. 1H). This suggests that at E5.5, BMP4 plays a major role in initiating BMP signaling activity in the mouse, and that this function of BMP4 is dose-dependent.

### Maternal *Bmp4* and *Smad4* are not required for development

Previous knockout studies of BMP signaling components did not report preimplantation phenotypes (Beppu et al., 2000; Mishina et al., 1995; Sirard et al., 1998; Winnier et al., 1995). However, other groups reported defects in preimplantation lineage specification using pathway inhibitors or microinjection of RNAi or mRNA for dominant-negative overexpression (Graham et al., 2014; Reyes de Mochel et al., 2015; Stuart et al., 2019). These observations raise the hypothesis that components of the BMP pathway are maternally imparted to the oocyte and functionally complement zygotically expressed components during early development. To test this hypothesis, we examined cell fate specification in embryos lacking both maternal (m) and zygotic (z) *Bmp4* or *Smad4* using the female germ line-expressed *Zp3*-*Cre* (De Vries et al., 2000) in combination with floxed alleles of either *Bmp4* or *Smad4* (see Fig. S3A for breeding scheme). RT-qPCR analysis confirmed the absence of detectable *Smad4* transcript in *Smad4* mz null oocytes (Fig. S3B), as we have observed for many other loci deleted in this manner (Blij et al., 2012; Frum et al., 2013, 2018; Wicklow et al., 2014).

**Fig. 2.**
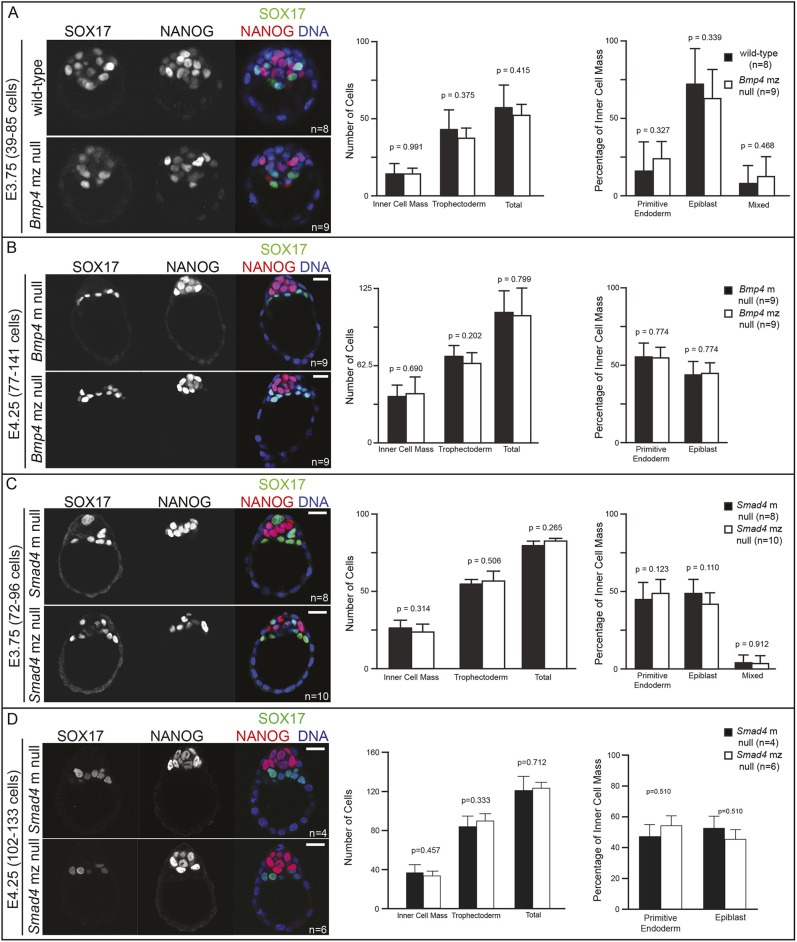
**Maternal and zygotic *Smad4* and *Bmp4* are dispensable for blastocyst formation and preimplantation cell fate specification.** (A) Immunofluorescence for SOX17 and NANOG as respective markers of primitive endoderm (PrE) and epiblast (EPI) in flushed E3.75 wild-type CD-1 embryos and embryos lacking maternal and zygotic *Bmp4* (mz null). Quantification did not reveal any significant difference in cell number or cell fate between *Bmp4* mz null embryos and controls. (B) Immunofluorescence for SOX17 and NANOG in flushed E4.25 embryos lacking maternal *Bmp4* only (m null) and *Bmp4* mz null embryos. Quantification did not reveal any significant difference in cell number or cell fate between *Bmp4* mz null embryos and controls. (C) Immunofluorescence for SOX17 and NANOG in flushed E3.75 *Smad4* m null and *Smad4* mz null embryos. Quantification did not reveal any significant difference in cell number or cell fate between *Smad4* mz null embryos and controls. (D) Immunofluorescence for SOX17 and NANOG in flushed E4.25 *Smad4* m null and *Smad4* mz null embryos. Quantification did not reveal any significant difference in cell number or cell fate between *Smad4* mz null embryos and controls. ‘Mixed’ indicates co-expression of SOX17 and NANOG. All pairwise comparisons were assessed by unpaired two-tailed Student's *t*-test. Error bars represent s.d. Scale bars: 10 μm.

Remarkably, we were able to recover *Bmp4* mz null and *Smad4* mz null blastocysts at wild-type rates (Fig. 2), indicating that neither *Bmp4* nor *Smad4* is required for either fertilization or preimplantation embryo development. Moreover, both *Bmp4* and *Smad4* mz null embryos exhibited normal morphology, total cell number, and ratio of trophectoderm and ICM cells (Fig. 2; Fig. S3C-E). Moreover, expression of the ICM marker OCT4 and polarized distribution of CDH1 in the TE strongly suggested normal lineage specification (Fig. S4). Similarly, the expression of EPI and PrE cell fate markers at E3.75, E4.25 and E4.5 was also unaffected in either *Bmp4* or *Smad4* mz null embryos at these stages (Fig. 2; Fig. S3C-E), consistent with normal ICM differentiation. Our observations support the conclusion that canonical BMP signaling does not play a major role in preimplantation development. In a parallel set of experiments, we allowed *Smad4* m null embryos to develop to term. Mice lacking maternal *Smad4* were born and developed apparently normally to at least 4 months old (15/15 mice, three litters). We conclude that maternal *Bmp4* and *Smad4* are dispensable for development and that neither zygotic gene plays any obvious role before implantation.

### *Smad4* promotes epiblast cavitation in a *Bmp4*-independent manner at E5.5

**Fig. 3.**
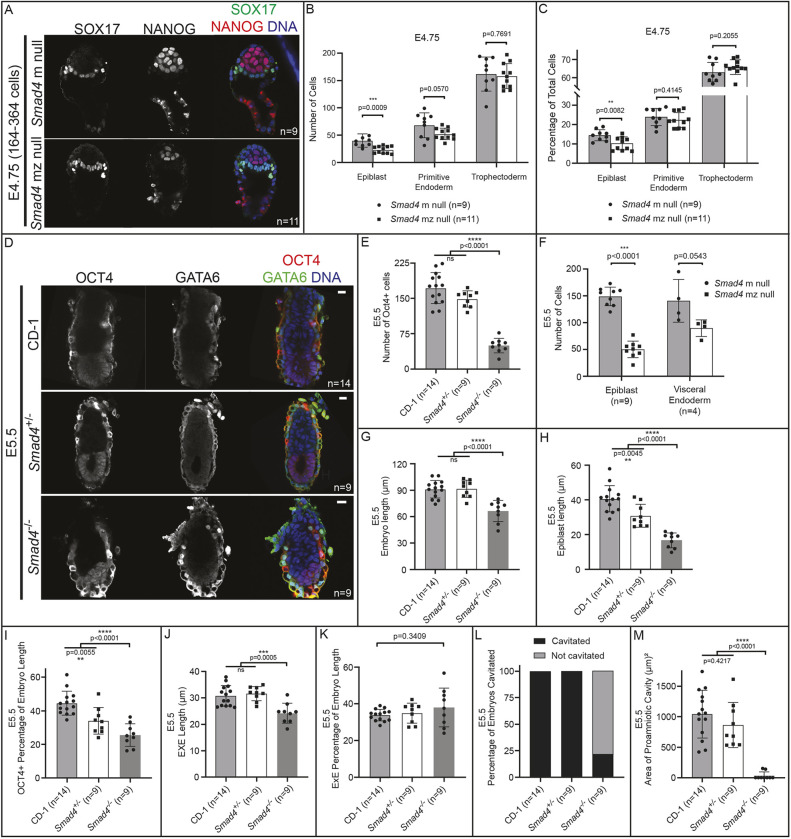
**BMP-independent function of *Smad4* is required for post-implantation epiblast organization and maintenance.** (A) E4.75 *Smad4* mz null and m null embryos stained by immunofluorescence for SOX17 and NANOG. (B) Quantification of epiblast (EPI), primitive endoderm (PrE) and trophectoderm (TE) cell numbers from embryos in A revealed a significant decrease in EPI cells in *Smad4* mz null embryos when compared with controls. (C) Quantification of the EPI, PrE and TE cells as a percentage of total cell number from embryos in A revealed a significant decrease in EPI percentage in *Smad4* mz null embryos. (D) E5.5 *Smad4*^−/−^ embryos stained by immunofluorescence for OCT4 and GATA6 as markers of EPI and VE, respectively. *Smad4*^−/−^ refers to combined *Smad4* z null and *Smad4* mz null embryos. (E) Quantification of the number of OCT4-positive cells in wild-type, *Smad4*^+/−^ and *Smad4*^−/−^ embryos. (F) Quantification of EPI and PrE cell numbers from *Smad4*^+/−^ and *Smad4*^−/−^ embryos at E5.5 revealed a specific, significant decrease in EPI cell number in *Smad4* mz null embryos when compared with controls (*P*<0.05, unpaired two-tailed Student's *t*-test). The difference in VE cell numbers was not significant (*P*>0.05). (G) Quantification of the proximal-distal length of wild-type, *Smad4*^+/−^ and *Smad4*^−/−^ embryos at E5.5. (H) Quantification of the proximal-distal length of the EPI of wild-type, *Smad4*^+/−^ and *Smad4*^−/−^ embryos at E5.5. (I) Quantification of the proximal-distal length of the EPI as a percentage of total length of wild-type, *Smad4*^+/−^ and *Smad4*^−/−^ embryos at E5.5. (J) Quantification of the proximal-distal length of the EXE of wild-type, *Smad4*^+/−^ and *Smad4*^−/−^ embryos at E5.5. (K) Quantification of the proximal-distal length of the EXE as a percentage of total length of wild-type, *Smad4*^+/−^ and *Smad4*^−/−^ embryos at E5.5. (L) Quantification of the proportion of *Smad4*^+/−^ and *Smad4*^−/−^ embryos with a proamniotic cavity at E5.5. (M) Quantification of the two-dimensional area of the proamniotic cavity of wild-type, *Smad4*^+/−^ and *Smad4*^−/−^ embryos at E5.5. Cavity area was measured within the plane exhibiting the largest cavity area for each embryo. Comparisons in B, C and F were assessed by unpaired two-tailed Student's *t*-test. Comparisons in E, G-K and M were assessed by analysis of variance (ANOVA) with Tukey's post-hoc test. **P<0.01, ***P<0.001, ****P<0.0001. ns, not significant. Data are mean±s.d. Scale bars: 10 μm.

Previous studies have mainly focused on characterization of BMP signaling loss-of-function phenotypes at later stages (>E5.5) (Sirard et al., 1998; Winnier et al., 1995; Yang et al., 1998). However, we first observed BMP signaling activity in most embryos just after implantation at E4.75, prompting us to scrutinize embryos lacking *Bmp4* or *Smad4* beginning at E4.75 for phenotypes. At E4.75, *Smad4* null embryos were grossly normal (Fig. 3A). However, upon close examination, *Smad4* null embryos displayed a significant decrease in total cell number (Fig. 3B). We quantified the number of EPI, PrE and TE cells in these embryos and discovered that the decreased cell number was most pronounced in EPI cells at this stage (Fig. 3B,C).

By E5.5, *Smad4* null embryos were visibly reduced in size and all displayed disorganization in EPI, VE and EXE compartments, as expected from studies performed at E6.5 (Sirard et al., 1998; Yang et al., 1998) (Fig. 3D). Strikingly, the EPI was greatly reduced in cell number (Fig. 3D-F) relative to controls, and had not yet cavitated, as the proamniotic cavity was not present among most (>80%, *n*=7/9) *Smad4* null embryos examined (Fig. 3D,L,M). Notably, relative to extra-embryonic lineages, the size of the EPI was disproportionately decreased in *Smad4* null embryos; the EPI length was decreased even when normalized to proximal-distal embryo length (Fig. 3G-I; Fig. S5A). By contrast, the size of the EXE was appropriately scaled to the reduced size of *Smad4* null embryos at E5.5 (Fig. 3J,K). This suggests that *Smad4* is not only required for general embryonic growth, but also specifically required for EPI growth or scaling relative to total embryo size. These phenotypes were not observed in E5.5 *Bmp4* null embryos, which did not differ from wild-type in morphology, embryo size, or EPI or PrE cell number (Fig. S5B-E), suggesting that additional signaling pathways act upstream of SMAD4 to promote EPI growth and morphogenesis at this stage.

### Epiblast cavitation requires SMAD4-dependent inhibition of FGF/ERK signaling

**Fig. 4.**
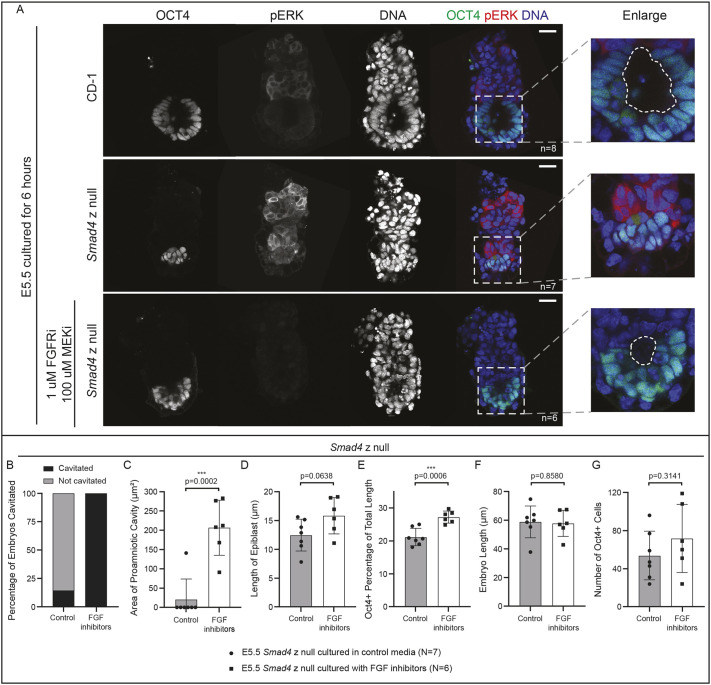
**Inhibition of FGF signaling partially rescues epiblast cavitation in E5.5 *Smad4* null embryos.** (A) Wild-type (CD-1) and *Smad4* z null embryos collected at E5.5 and cultured for 6 h after dissection in control media or media containing FGFR/MEK inhibitors (see Materials and Methods), then stained by immunofluorescence for OCT4 and phosphorylated ERK (pERK). Dashed line in enlargement denotes the proamniotic cavity. (B) Quantification of the proportion of treated and untreated *Smad4*^−/−^ embryos with a proamniotic cavity at E5.5. (C) Quantification of the two-dimensional area of the proamniotic cavity of treated and untreated *Smad4*^−/−^ embryos at E5.5. Cavity area was assessed on the *z*-plane with the largest cavity space for each embryo. (D) Quantification of proximal-distal length of the epiblast (EPI) in treated and untreated E5.5 *Smad4*^−/−^ embryos. (E) Quantification of proximal-distal length of the EPI as a proportion of total length in treated and untreated E5.5 *Smad4*^−/−^ embryos. (F) Quantification of proximal-distal length in treated and untreated E5.5 *Smad4*^−/−^ embryos. (G) Quantification of OCT4-positive cell number in treated and untreated E5.5 *Smad4*^−/−^ embryos. ***P<0.001. Comparisons in C-G were assessed using unpaired two-tailed Student's *t*-test.. Data are mean±s.d. Scale bars: 10 μm.

Having discovered that *Smad4* is required for EPI cavitation and growth at E5.5, we began to investigate the mechanism. We were struck by the observation that the EXE appeared to be oversized, relative to the size of the EPI, in E5.5 *Smad4* null embryos. To confirm the identity of the EXE cells, we examined markers of EXE, including phosphorylated ERK (pERK) (Corson et al., 2003). In *Smad4* null embryos, we observed pERK throughout the EXE region (Fig. 4A), consistent with its identity as EXE. Notably, however, pERK levels were dramatically increased within the EXE relative to wild-type embryos (Fig. 4A). As pERK in the EXE is dependent on signaling by the fibroblast growth factor (FGF) pathway (Corson et al., 2003), we hypothesized that increased pERK was because of elevated FGF signaling in *Smad4* null embryos.

To test this hypothesis, we used a previously-published protocol to inhibit FGF signaling in embryos (Yamanaka et al., 2010), which effectively eliminated pERK in control embryos (Fig. S6A). Additionally, inhibition of FGF signaling partially rescued EPI defects in E5.5 *Smad4* null embryos (Fig. 4A). That is, we observed a significant increase in EPI size, as well as increased rates of cavitation in FGF-inhibited *Smad4* null embryos, relative to untreated *Smad4* null embryos (Fig. 4B-E). However, FGF inhibitor treatment did not fully rescue the growth restriction of *Smad4* null embryos (Fig. 4F), nor was the number of OCT4-positive cells restored (Fig. 4G). These observations are consistent with SMAD4 limiting the level of FGF signaling within the EXE during early post-implantation stages to promote EPI morphogenesis.

To determine whether elevated FGF signaling is sufficient to antagonize cavitation, we treated embryos with exogenous FGF4. In wild-type E5.5 embryos treated with exogenous 1 µg/ml FGF4, we observed elevated levels of pERK within the EXE and ectoplacental cone (Fig. S6A). However, we observed no impact on EPI cavitation or EPI size following this treatment (Fig. S6A-D). Altogether, these data suggest that pERK/FGF signaling antagonizes EPI cavitation, but upregulation of pERK alone is insufficient to induce cavitation defects in wild-type embryos, at least under the conditions tested here.

## DISCUSSION

Here, we have used a combination of immunofluorescence and genetic approaches to elucidate the roles of BMP4, pSMAD1/5/9 and SMAD4 in peri-implantation development. We have considered why our preimplantation pSMAD1/5/9 findings differ from previously published findings (Graham et al., 2014; Reyes de Mochel et al., 2015), and we have identified several differences in experimental design that could be relevant. The first difference is the source and specificity of antibodies. We used the same anti-pSMAD1/5/9 antibody that was used in post-implantation embryos in Senft et al. (2019), producing concordant post-implantation results. However, a different antibody was used to detect pSMAD1/5/9 in preimplantation embryos in Reyes de Model et al. (2015). The source of antibody that was used to detect pSMAD1/5/9 in blastocysts in Graham et al. (2014) was not described in detail, but a more recent study from the same lab reported absence of pSMAD1/5/9 in the blastocyst using the same antibody that we used (Weatherbee et al., 2024). Ours is the only study to confirm pSMAD1/5/9 specificity in *Bmp4* null blastocysts, providing strong evidence for antibody specificity.

A second possible difference between our study and previous studies is embryo preparation: we characterized pSMAD1/5/9 localization in embryos immediately after retrieval from pregnant mice, whereas embryos were cultured before analysis in Graham et al. (2014). Moreover, embryos were cultured in the presence of bovine serum albumin (BSA) in Graham et al., which could influence the signaling environment of the embryo. A third possible difference is the loss-of-function approach: previous studies have primarily modulated BMP signaling using either pharmacological inhibitors or overexpression of dominant-negative receptors (Graham et al., 2014; Reyes de Mochel et al., 2015). It is therefore possible that the previously reported preimplantation phenotypes were due to expression of proteins at super-physiological levels and/or off-target effects of chemical inhibitors. Consistent with this latter proposal, the BMP inhibitors dorsomorphin, LDN-193189 and DMH2 have been shown to inhibit dozens of kinases that are not members of the BMP signaling pathway (Boergermann et al., 2010; Lowery et al., 2016; Vogt et al., 2011). This possibility also complicates interpretation of post-implantation embryo phenotypes resulting from treatment with BMP signaling inhibitors (Sozen et al., 2021).

Here, we also showed that pSMAD1/5/9 is first detectable within the PrE lineage. As *Bmp4* is expressed in EPI cells at the blastocyst stage, and in EXE during post-implantation stages, and as our data show that SMAD1/5/9 phosphorylation in the PrE lineage is *Bmp4*-dependent, our observations indicate that BMP4 signals to the PrE lineage non cell-autonomously. Yet, the role for BMP4-SMAD1/5/9-SMAD4 in the PrE lineage is unclear. Intriguingly, BMP4 is sufficient to induce differentiation of PrE-derived extra-embryonic endoderm stem (XEN) cells to VE-like cells (Artus et al., 2012; Paca et al., 2012). Therefore, BMP4 signaling via pSMAD1/5/9 may facilitate PrE maturation *in vivo*.

Our observations additionally suggest that the spatial patterning of BMP signaling is limited in part by the availability of the ligand during early postimplantation. Consistent with this, the range of SMAD1/5/9 phosphorylation could be expanded within the VE by addition of exogenous BMP4. Curiously, however, we did not detect pSMAD1/5/9 within the EXE or proximal EPI of wild-type embryos, suggesting that additional mechanisms exist for limiting BMP signaling to the EXE-adjacent VE. We do not yet know whether this pattern is important for development.

**Fig. 5.**
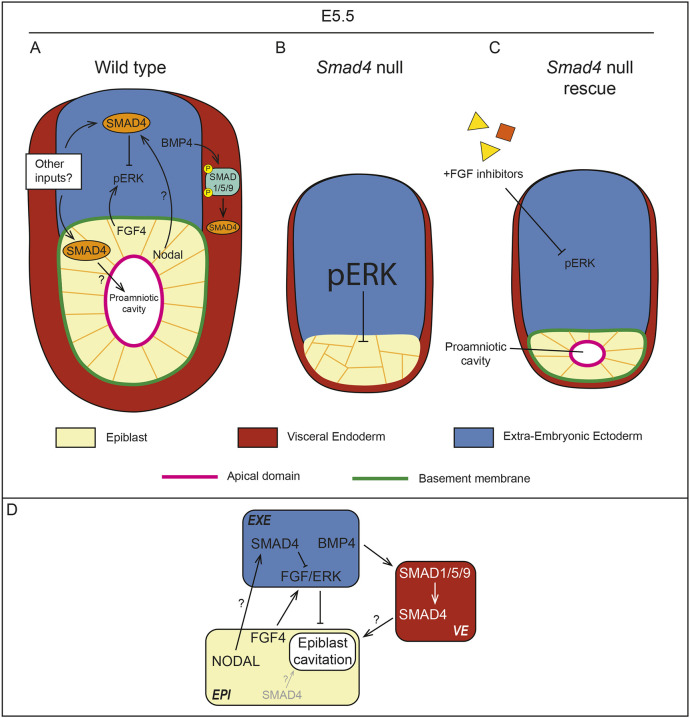
**Working model: FGF inhibition rescues rosette formation but not embryo growth in *Smad4* null embryos.** (A) In wild-type embryos, SMAD4 activity inhibits ERK phosphorylation in the extra-embryonic ectoderm (EXE), which allows for rosette formation and cavitation in the epiblast (EPI). BMP4 from the EXE activates SMAD1/5/9 phosphorylation and SMAD4 activity in the visceral endoderm (VE), but this activity is not required for EPI cavitation. (B) In *Smad4* null embryos, pERK is upregulated, causing an increase in pERK in the EXE and preventing EPI cavitation. (C) Treatment with FGF inhibitors prevents ectopic upregulation of pERK in *Smad4* null embryos, resulting in a small proamniotic cavity. (D) Proposed mechanism for regulation of EPI cavitation by SMAD4.

Our study demonstrates a requirement for *Smad4* in EPI maintenance and morphogenesis postimplantation (Fig. 5). We have considered whether these findings differ from previous reports. Previous descriptions of *Smad4* null embryos report growth restriction and VE disorganization beginning at E5.5, with embryonic lethality by E8.5 (Chu et al., 2004; Sirard et al., 1998; Yang et al., 1998). Our results are consistent with these findings, but also uncover previously unappreciated defects in the EPI. Notably, cavitation defects were not reported for E6.5 embryos homozygous for null or deleted alleles of *Smad4* (Sirard et al., 1998; Yang et al., 1998) (exon 8 deleted in both; Yang et al., allele used in the present study). However, E5.5 embryos were not examined in detail in these previous studies. Alternatively, genetic background, which is known to influence the severity of the *Bmp4* null phenotype (Winnier et al., 1995) could impact the severity or the penetrance of the *Smad4* EPI phenotypes. Both previous studies were performed in a C57BL/6 background, whereas ours were performed in a CD-1 background, where the cavitation phenotype was partially penetrant (80%).

Regardless of the explanation, our observations prompt us to wonder where *Smad4* is required for early EPI morphogenesis (E4.75-E5.5). A previous study characterized the spatial requirements for *Smad4* in embryogenesis by performing VE-specific *Smad4* deletion, which caused gastrulation defects (Chu et al., 2004)*.* These observations led to the conclusion that SMAD4 acts non cell-autonomously within the VE. However, the VE-specific Cre driver used to delete *Smad4* in this study is not active until E5.75, which is well after we first observe EPI defects. As our study first observed an EPI defect at E4.75, an earlier knockout of *Smad4* within the PrE lineage is needed to determine whether SMAD4 promotes EPI morphogenesis non cell-autonomously via the VE. On the other hand, our study also showed that EPI cavitation relies on SMAD4 attenuation of pERK levels in the EXE, strongly suggesting that SMAD4 promotes EPI cavitation non cell-autonomously via the EXE. Regrettably, distinguishing between these two intriguing possibilities is not experimentally straightforward at the current time.

We note that we have not ruled out a cell-autonomous requirement for *Smad4* within the EPI. For example, SMAD4 could promote EPI maturation, defined here as the transition from a naïve to primed state, which normally occurs in EPI cells between preimplantation and post-implantation stages (Boroviak et al., 2015; Nichols and Smith, 2009). EPI maturation has been suggested to be crucial in the formation of the proamniotic cavity (Carbognin et al., 2023; Shahbazi et al., 2017). The possible cell-autonomous requirement for *Smad4* is not mutually exclusive with its possible non cell-autonomous roles in the extra-embryonic lineages.

At this point, we understand little about the signals acting upstream and downstream of SMAD4 in EPI cavitation and scaling (E4.75-E5.5). In terms of upstream signals, a previous study has reported that BMP2/4 signaling is necessary and sufficient for cavitation of embryoid bodies (Coucouvanis and Martin, 1999). If BMP4 is functionally redundant with BMP2 or other BMP factors in this context, this could explain why cavitation appeared to be normal in E5.5 *Bmp4* null embryos. However, we do not favor this model because phosphorylation of SMAD1/5/9 was dependent on *Bmp4*, arguing against functional redundancy with other BMP ligands. Deletion of genes encoding all three SMAD1/5/9 factors would be necessary to test the requirements for BMP signaling in early EPI morphogenesis.

Alternatively, another TGFβ pathway may signal through SMAD4 to regulate early EPI morphogenesis. Knockout of *Nodal* has been shown to decrease embryo size and expression of *Oct4* mRNA at early post-implantation stages (Brennan et al., 2001; Mesnard et al., 2006), but *Nodal* null embryos cavitate normally and expression of OCT4 protein is normal at E5.5 (Senft et al., 2019). As the cavitation defect and loss of OCT4-positive cells are more severe in *Smad4* null embryos than either *Bmp4* null or *Nodal* null models alone, multiple TGFβ pathways may regulate EPI morphogenesis cooperatively (Fig. 5).

We also do not yet understand the mechanisms regulating early EPI morphogenesis downstream of SMAD4. We showed that SMAD4 promotes cavitation by attenuating pERK levels. Inhibition of FGF/MAPK signaling rescued cavitation in *Smad4* null embryos, even though the EPI remained disproportionately small. This observation allows us to propose that cavitation is not dependent on EPI size, but rather the embryo signaling environment. Identification of SMAD4 targets in the EXE using an approach such as CUT&RUN could help reveal the mechanism by which SMAD4 regulates ERK levels in the EXE. Further study will also be needed to discover previously unreported signals from the EXE that act downstream of ERK to promote EPI morphogenesis.

## MATERIALS AND METHODS

### scRNA-seq analysis

ScRNA-seq data generated by Nowotschin et. al. was used to analyze the expression of TGFβ genes in mouse E3.5, E4.5, E5.5 and E6.5 blastocysts (Nowotschin et al., 2019). The analysis was completed using R v4.1.0 with tools from Seurat v4.3.0 (Hao et al., 2021). We normalized the UMI counts using SCTransform and cells were visualized in 2D space using UMAP performed on the first 30 principal components (Choudhary and Satija, 2022; Hafemeister and Satija, 2019). After excluding TGFβ genes expressed in <10 cells, we used Seurat's FindAllMarkers function with the Wilcoxon rank-sum test to identify TGFβ genes enriched in each cell type versus all other cells. The *P*-values were corrected for multiple comparisons using the Bonferroni method. Genes with *P*-adj<0.01 and average log_2_ fold change<0.25 were considered cluster enriched. Heatmaps were generated using the pheatmap (v 1.0.12) after averaging the normalized expression for each gene in each cell type.

### Mouse strains and genotyping

All animal research was conducted in accordance with the guidelines of the Michigan State University Institutional Animal Care and Use Committee. Wild-type embryos were derived from CD-1 mice (Charles River). The following alleles were used in this study and maintained in a CD-1 background (see Table S1): *Bmp4^tm1Jfm^/J* (Liu et al., 2004); *Smad4^tm2.1Cxd^/J* (Yang et al., 2002); *Tg(Zp3-cre)93Knw* (de Vries et al., 2000). Null alleles were generated by breeding dams carrying homozygous floxed alleles and the *Zp3Cre* allele to CD-1 males. Mouse genotypes were determined by PCR using genomic DNA extracted using the REDExtract-N-Amp kit (Sigma-Aldrich, XNAT) according to the manufacturer's protocol. Embryo genomic DNA was extracted using the same kit scaled to 10 µl total volume. Genomic extracts (1-2 µl) were then subjected to PCR using allele-specific primers (see Table S3).

### Embryo collection and culture

Mice were maintained on a 12 h light/12 h dark cycle. Preimplantation (E2.5-E4.5) embryos were collected by flushing the oviduct or uterus with M2 medium (Sigma-Aldrich, M7167). Post-implantation (E4.75-E6.5) embryos were collected by dissecting the embryos from the decidua in ice-cold PBS containing 1% fetal bovine serum (FBS; HyClone, SH30396.02) or BSA (Sigma-Aldrich, A7888). During embryo collection, dissected embryos were held in warm M2 media. For embryo culture, KSOM medium (Millipore, MR-121-D) was equilibrated overnight before embryo collection. Where indicated, the following were included in the culture medium: 1 µM or 0.25 µM LDN-193189 in DMSO (Stemgent, 04-0074-02); 1 µg/ml recombinant FGF4 in PBS with 0.1% BSA (R&D Systems, 235-F4); 1 µg/ml heparin (Sigma-Aldrich, H3149); 100 ng/ml recombinant BMP4 in 4 mM HCl (R&D Systems, 314-BP); 1 µM PD173074 in DMSO (FGFRi, Selleckchem, S1264); 5 µM PD0325901 in DMSO (MEKi, Stemgent, 04-0006); or 0.2% DMSO (New England BioLabs, B0515A) as control. Embryos were cultured at 37°C in a 5% CO_2_ incubator under light mineral oil (Millipore, ES-005-C).

### Real-time PCR of oocytes

*Smad4* expression levels in oocytes were assessed by real-time PCR as previously described (Blij et al., 2012). *Smad4* levels were assessed in oocytes from three wild-type and three *Smad4* maternal null females. Oocytes collected from each female were pooled for mRNA extraction and cDNA synthesis. RT-PCR was performed in quadruplicate technical replicates for each cDNA sample. Primers were (5′-3′): *Actb*, CTGAACCCTAAGGCCAACC and CCAGAGGCATACAGGGACAG; *Smad4* (wild-type allele), CGCGGTCTTTGTACAGAGTTA and ACACTGCCGCAGATCAAAG; *Smad4* (deleted allele), CACAGGACAGAAGCGATTGA and CCAAACGTCACCTTCACCTT.

### Immunofluorescence and confocal microscopy

Preimplantation embryos (E2.5-E4.75) were fixed with 4% formaldehyde (Polysciences, 04018) for 10 min, permeabilized with 0.5% Triton X-100 (Sigma-Aldrich, X100) for 30 min, and then blocked with blocking solution (10% FBS, 0.1% Triton X-100) overnight at 4°C. Embryos were incubated with primary antibody overnight at 4°C. The next day, embryos were washed in blocking solution for 30 min, incubated in secondary antibody diluted in blocking solution for 1 h, washed in blocking solution for 30 min, then stained with nuclear stain (DRAQ5) diluted in blocking solution for 10 min or overnight.

Post-implantation embryos (E5.0-E5.75) were fixed with 4% formaldehyde for 1 h, washed three times in 0.1% Tween-20 (Sigma-Aldrich, P9416), permeabilized for 4 h in 0.5% Triton X-100, and then blocked with blocking solution [3% BSA (Sigma-Aldrich, A7888); 0.3% Triton X-100 in PBS] overnight at 4°C. Embryos were incubated with primary antibody overnight at 4°C. The next day, embryos were washed three times in 0.1% Tween-20 for 5 min, then incubated in secondary antibody diluted in blocking solution overnight. The following day embryos were washed three times in 0.1% Tween-20 for 5 min, then stained with nuclear stain diluted in blocking solution for 10 min or overnight.

All embryos (preimplantation or postimplantation) which used antibodies against pSMAD1/5/9 were fixed with 4% formaldehyde for 1 h, methanol dehydration-rehydration series (25%, 50%, 75%, 100%) for 5 min each, washed three times in freshly-made 1% Triton X-100 for 10 min, washed for 20 min in ice-cold acetone at −20°C, washed three times in freshly-made 1% Triton X-100 for 10 min, then then blocked with blocking solution (10% FBS, 0.1% Triton X-100 in PBS) overnight at 4°C. Embryos were incubated with primary antibody overnight at 4°C. The next day, embryos were washed three times in freshly prepared 0.1% Triton X-100 for 10 min, incubated in secondary antibody diluted in blocking solution for 2 h, washed three times in freshly-made 0.1% Triton X-100 for 10 min, then stained with nuclear stain diluted in blocking solution for 10 min or overnight.

All embryos (preimplantation or postimplantation) which used antibodies against pERK were fixed with 4% formaldehyde for 1 h, washed three times for 5 min in PBS, washed for 20 min in ice-cold methanol at −20°C, permeabilized for 30 min in 0.1% Tween-20, then blocked with blocking solution (3% BSA; 0.3% Triton X-100 in PBS) overnight at 4°C. Embryos were incubated with primary antibody overnight at 4°C. The next day, embryos were washed three times in PBS for 5 min, incubated in secondary antibody diluted in blocking solution for 2 h, washed three times in PBS for 5 min, then stained with nuclear stain diluted in blocking solution for 10 min or overnight. All solutions contained HALT protease inhibitor (Thermo Fisher Scientific, 78430) and PhosSTOP phosphatase inhibitor (Roche, 04906837001) diluted 1:500.

Antibodies used are listed in Table S2. Embryos were imaged using an Olympus FluoView FV1000 Confocal Laser Scanning Microscope system with 60× PlanApoN oil (NA 1.42) objective. For each embryo, *z*-stacks were collected, with 5 µm intervals between optical sections. All embryos were imaged before knowledge of their genotypes.

### Embryo analysis

For each embryo, *z*-stacks were analyzed using Fiji (ImageJ), which enabled the labeling, based on DNA stain, of all individual cell nuclei. Using this label to identify individual cells, each cell in each embryo was then assigned to relevant phenotypic categories, without knowledge of embryo genotype. Phenotypic categories included marker expression (e.g. OCT4-positive or -negative) and marker localization (e.g. pSMAD1/5/9 nuclear, absent or unlocalized). Proamniotic cavity area was measured using Fiji. Statistical analysis was performed using GraphPad Prism (v. 9.5.1). Comparisons between two groups were performed using unpaired two-tailed Student's *t*-test, where *P*<0.05 was considered significant. Comparisons between three or more groups were performed using analysis of variance (ANOVA) followed by Tukey's post-hoc, where *P*<0.05 was considered significant. Figure images were assembled using Adobe Illustrator.

## Supplementary Material

10.1242/develop.202377_sup1 Supplementary information
